# Identification and genomic characterization of a novel species of feline anellovirus

**DOI:** 10.1186/s12985-016-0601-8

**Published:** 2016-08-27

**Authors:** Wen Zhang, Hua Wang, Yan Wang, Zhijian Liu, Jingjiao Li, Lianghua Guo, Shixing Yang, Quan Shen, Xiaoying Zhao, Li Cui, Xiuguo Hua

**Affiliations:** 1 School of Medicine, Jiangsu University, 301 Xuefu Road, Zhenjiang, Jiangsu 212013 People’s Republic of China; 2 School of Agriculture and Biology, Shanghai Jiaotong University, 800 Dongchuan Road, Shanghai, 200240 China

**Keywords:** Cat, Anellovirus, Complete genome, Viral metagenomics, Phylogenetic analysis

## Abstract

Here, a novel feline anellovirus strain (named FelineAV621 and GenBank no. KX262893) was detected in two cats with diarrhea. The complete genome of FelineAV621 is 2409 nt long with a G+C content of 56.67 %, including three open reading frames (ORFs). Phylogenetic analysis based on the amino acid sequence of the putative capsid protein (ORF1) indicated that FelineAV621 belonged to a novel anellovirus species inside a clade containing the seal anellovirus, canine TTVs, and porcine TTVs, but was distant from all the previous feline anelloviruses.

## Findings

Anelloviruses belong to non-enveloped, circular, single-stranded DNA viruses with genome of 2.1–3.9 kb in length depending on the isolate analyzed [1]. Anelloviruses are subgrouped into Torque teno virus (TTV), Torque teno mini virus (TTMV), Torque teno midi virus (TTMDV) and small anellovirus (SAV) [2]. Although anelloviruses are widely prevalent in humans and animals and suspected to be associated with some diseases of humans [3–6] or animals [7–9], the etiological role of anelloviruses has not yet been clearly identified.

The first report of discovering feline anellovirus was published by Okamoto et al. [10] and subsequently reported in several other publications [11–14]. Phylogenetic analysis based on the amino acid sequences of ORF1 of feline anelloviruses available in GenBank revealed that these known feline anellovirus clustered together and were classified into two species, Torque teno felis virus 1 (FcTTV1) and Torque teno felis virus 2 (FcTTV2), belonging to the genus Etatorquevirus within the family Anelloviridae [13]. The aim of this study is to characterize the genome sequence of a divergent feline anellovirus which is present in the fecal and blood samples from domestic cats with diarrhea.

In a previous study, where viral metagenomic method was used to investigate the fecal virome of cats in a shelter in California, USA, several sequencing reads showed amino acid similarity to viruses in the family *Anelloviridae* [14]. In this study, nested primers designed based on a 621 bp contig assembled by geneious software from the data of that study [14] are used to investigate whether this anellovirus is present in cat population in China. The primers used here are Anel621FO (5′- ACTTCCTCCTGGTCGGGCGT-3′) and Anel621RO (5′- TGGGGAGGGGTTGATGCCCA-3′) for the 1st round PCR, and Anel621FI (5′- GGCTCTCGTGCGGTTTGGGA-3′) and Anel621RI (5′- TCCCGTCGTCCCACCACCAT-3′) for the 2nd round PCR. The PCR product size of the 2nd round PCR is about 215 bp. PCR screening was performed to detect the anellovirus-like sequence in a total 22 samples, including 11 blood and 11 fecal samples collected from 11 cats with diarrhea from Jan. 2014 to Dec. 2015. Results indicated that two blood samples and one fecal samples were positive, including one cat positive both in blood and fecal samples, and one positive blood sample. The specific DNA band was T-A cloned and sequencing result indicated that the two sequences from blood and fecal samples of the same cat were identical and had one nucleotide difference from the sequence from the other blood sample in the present study. Sequence analysis indicated that the two sequences from cats in the present study shared 97.7 % sequence identity with the sequences from the California cats [14], with five different nt over the 215 bp sequence fragment.

To sequence the entire genome of the feline anellovirus, the viral nucleic acid was extracted form one of the cat the blood samples. Circular viral DNA molecules were preferentially amplified by rolling circle amplification (RCA) using random hexamer primers and the Illustra TempliPhi amplification kit (GE Healthcare). Outwardly pointing specific PCR primers FelAVF (5′-ATGCTCAACACCACAAACGC-3′) and FelAVR (5′-TGTAATCCCAAACCGCACGA-3′) were designed from the viral metagenomic sequence [14] and then used to amplify the small circular genomes by inverse PCR. The PCR products were then sequenced by primer walking and Sanger sequencing.

**Fig. 1:**
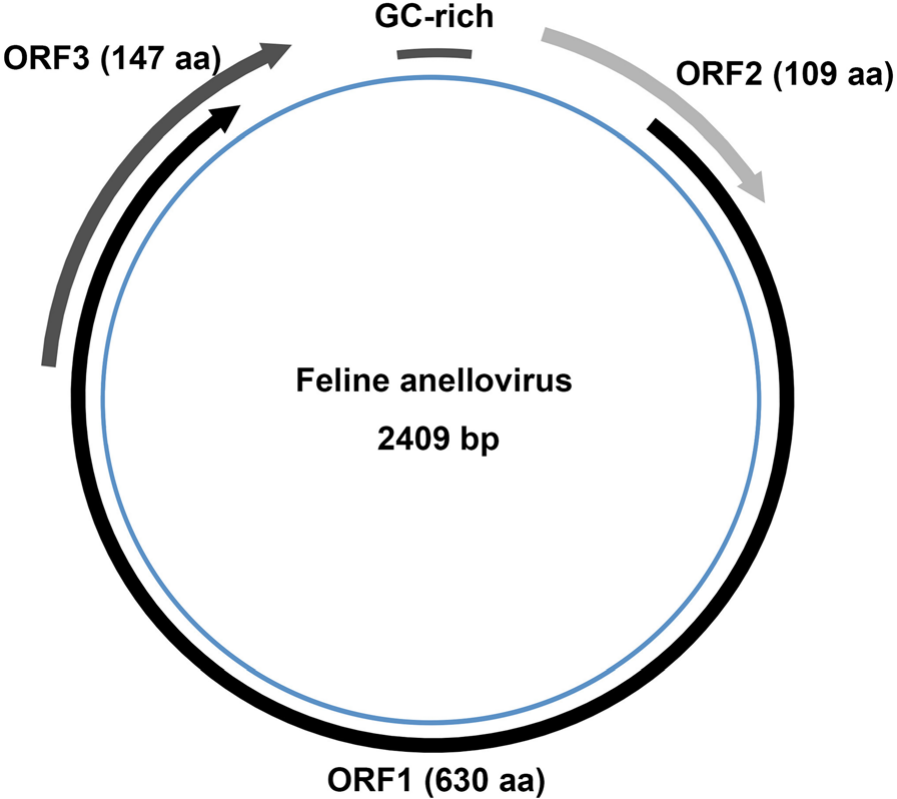
Genomic organization of the feline anellovirus (named FelineAV621 and GenBank no. KX262893)

Sequencing results indicated that the complete genome of the feline anellovirus strain (named FelineAV621 and GenBank no. KX262893) was 2409 nt long, with a G+C content of 56.67 %. The genome organization of FelineAV621 is consistent with that of other anelloviruses, containing three open reading frames (ORFs) (Fig. 1). The ORF1, the largest ORF, does not display any significant nucleotide identity to any virus sequence using BLASTn in GenBank, but shares low identity with a seal anellovirus (GenBank no.: KM262782) (30.2 % identity) [15] and a pine marten torque teno virus (GenBank no.: JN704611) (29.4 % identity) [16] based on the amino acid sequence of ORF1. ORF1 encodes a 630 amino acid long putative capsid protein which presents three potential glycosylation sites; no signal peptides were identified. As in other anelloviruses, the capsid protein is arginine rich in its N terminus, with 31 arginine residues in the first 60 amino acids (51.67 %). ORF2 encodes 109 amino acids and shares 29–40 % amino acid sequence identities with several ORF2 proteins of anelloviruses in GenBank. ORF3 encodes147 amino acids and shares no amino acid sequence identity with proteins in GenBank.

**Fig. 2:**
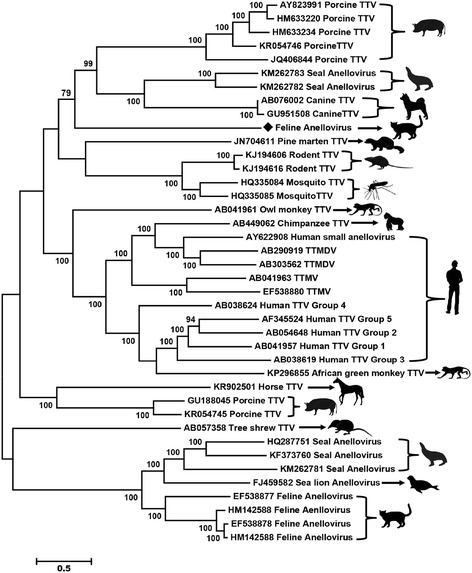
Phylogenetic tree based on the full-length amino acid sequence of ORF1 depicting relationships among the members of the family *Anelloviridae*. The newly discovered feline anellovirus is indicated by a diamond shape. The host is indicated in animal profiles and GenBank accession numbers are shown

To determine the relationship of FelineAV621 to other anelloviruses, phylogenetic analysis was performed based on the ORF1 amino acid sequences of FelineAV621, its best BLASTp matches in GenBank and the representative members of related viruses. Sequence alignment was performed using CLUSTALW with the default settings. A phylogenetic tree with 1000 bootstrap resamples of the alignment data sets was generated using the maximum likelihood method based on Jones-Taylor-Thornton (JTT) model in MEGA5.0. The anellovirus tree topology was consistent with results of earlier studies [1, 9, 17] and revealed that FelineAV621 is a novel anellovirus species that belongs to a clade that contains the seal anellovirus, canine TTVs, and porcine TTVs, but distant from the previous four feline anelloviruses (Fig. 2), suggesting cat can carry at least two different species of anelloviruses.

Taken together, a novel species of feline anellovirus was detected in two cats with diarrhea, including both blood and fecal samples of one cat and the blood sample of the other one. The complete genome of this novel anellovirus was sequenced and characterized from one of the blood sample. Phylogenetic analysis revealed that this feline anellovirus belonged to a novel anellovirus species which is significantly divergent from the previous feline anellovirus strains. Due to the limited sample number and lacking serologic evidence in the present study, whether FelineAV621 can really infect cats and cause diarrhea will require further epidemiologic study based on a larger sample size and testing cat sera for specific antibodies.

## Abbreviations

BLAST: Basic local alignment search tool
ORF: Open reading frame
PCR: Polymerase chain reaction
SAV: Small anellovirus
TTMV: Torque teno mini virus, TTMDV, Torque teno midi virus
TTV: Torque teno virus
